# Supplementary material for: Evidence-based design and evaluation of a whole genome sequencing clinical report for the reference microbiology laboratory
Source: PeerJ. 2018 Jan 10;6:e4218. doi: 10.7717/peerj.4218 (PMC5767084; doi:10.7717/peerj.4218)
Supplement: Supplemental Information 1 [file peerj-06-4218-s001.pdf]

Supplemental Material for

Evidence-Based Design and Evaluation of a Whole Genome Sequencing Clinical Report for the Reference Microbiology Laboratory

Anamaria Crisan, Geoffrey McKee, Tamara Munzner, and Jennifer L. Gardy

Contents

1. Supplemental Tables S1-S6
2. Supplemental Figure S1
3. Survey Instrument 1: Task and Data Questionnaire
4. Survey Instrument 2: Design Choices Questionnaire
5. Final Design Walkthrough
6. ISO15189 Requirements

Table S1. Task and Data Questionnaire respondents' self-reported training levels.

	Training Level				
Subject Area	None	Undergrad.	Graduate/ Medical Training*	Professional Experience**	Continuing Education***
Molecular Biology, Biochemistry	29.4%	29.4%	47.1%	41.2%	35.3%
Epidemiology	11.8%	5.9%	58.5%	64.7%	41.2%
Biostatistics	58.8%	11.8%	29.4%	23.5%	23.5%
Bioinformatics	52.9%	0.0%	11.8%	35.3%	29.4%
Genomics	23.5%	5.9%	23.5%	47.1%	52.0%
Infectious Disease	5.9%	35.3%	58.8%	76.5%	52.9%
Respiratory Medicine	17.4%	1.4%	29.4%	47.1%	29.4%

Note: Participants could select one or more levels of training, thus, rows will not add to 100%
*Graduate includes Masters & PhD
*Professional experience such as collaborating with others on a project
**Continuing education such as attending workshops, training sessions, or self-directed learning

Table S2. Task and Data Questionnaire respondents' anticipated future use of molecular/genomic data.

Data Type	Extent of usage					
	Never	Rarely	Sometimes	Often	All the time	Don't know what this is
Patient information	1 (5.9%)	0 (0.0%)	1 (5.9%)	1 (5.9%)	14 (82.4%)	0 (0.0%)
Patient's own prior TB test result	0 (0.0%)	0 (0.0%)	3 (17.6%)	1 (5.9%)	12 (70.6%)	1 (5.9%)
Requester identifier	2 (11.8%)	2 (11.8%)	2 (11.8%)	2 (11.8%)	9 (52.9%)	0 (0.0%)
Review identifier	2 (11.8%)	2 (11.8%)	4(23.5%)	0 (0.0%)	8 (47.1%)	1 (5.9%)
Type of sample	0 (0.0%)	0 (0.0%)	1 (5.9%)	5 (24.9%)	11 (64.7%)	0 (0.0%)
Sample collection site	0 (0.0%)	2 (11.8%)	0 (0.0%)	1 (5.9%0	11 (64.7%)	0 (0.0%)
Sample collection date	0 (0.0%)	0 (0.0%)	2 (11.8%)	2 (11.8%)	13 (76.5%)	0 (0.0%)
Interpretation or comments from reviewer	3 (17.6%)	2 (11.8%)	2 (11.8%)	1 (5.9%)	11 (64.7%)	0 (0.0%)
Tuberculin Skin Test (TST) results	4 (23.5%)	2 (11.8%)	2 (11.8%)	2 (11.8%)	7 (41.2%)	0 (0.0%)
Interferon Gamma Release Assay (IGRA) results	3 (17.6%)	2 (11.8%)	1 (5.9%)	4 (23.5%)	7 (41.2%)	0 (0.0%)
Chest X-ray	3 (17.6%)	2 (11.8%)	0 (0.0%)	3 (17.6%)	9 (52.9%)	0 (0.0%)
Acid Fast Bacilli (AFB) smear status	2 (11.8%)	1 (5.9%)	1 (5.9%)	1 (5.9%)	12 (70.6%)	0 (0.0%)
Culture results	1 (5.9%)	0 (0.0%)	0 (0.0%)	2 (11.8%)	14 (82.4%)	0 (0.0%)
Speciation	0 (0.0%)	0 (0.0%)	1 (5.9%)	0 (0.0%)	16 (94.1%)	0 (0.0%)
Phenotypic Drug Susceptibility Test (DST) results	0 (0.0%)	0 (0.0%)	1 (5.9%)	1 (5.9%)	15 (88.2%)	0 (0.0%)
Molecular DST results	0 (0.0%)	0 (0.0%)	1 (5.9%)	4 (23.5%)	12 (70.6%)	0 (0.0%)
Specific mutations conferring drug resistance	1 (5.9%)	0 (0.0%)	1 (5.9%)	5 (24.9%)	9 (52.9%)	1 (5.9%)
Spoligotype	3 (17.6%)	3 (17.6%)	1 (5.9%)	3 (17.6%)	2 (11.8%)	5 (29.4%)
MIRU-VNTR	0 (0.0%)	1 (5.9%)	1 (5.9%)	4 (23.5%)	11 (64.7%)	0 (0.0%)
RFLP	3 (17.6%)	6 (35.3%)	1 (5.9%)	2 (11.8%)	1 (5.9%)	4 (23.5%)
Cluster assignment	0 (0.0%)	2 (11.8%)	2 (11.8%)	1 (5.9%)	12 (70.6%)	0 (0.0%)
SNP distance from other isolates	1 (5.9%)	3 (17.6%)	1 (5.9%)	2 (11.8%)	9 (52.9%)	1 (5.9%)
Phylogenetic tree	1 (5.9%)	2 (11.8%)	3 (17.6%)	2 (11.8%)	6 (25.3%)	3 (17.6%)
Laboratory performance measures	2 (11.8%)	3 (17.6%)	1 (5.9%)	5 (24.9%)	5 (29.4%)	1 (5.9%)

Table S3. Task and Data Questionnaire respondents' confidence in their ability to interpret various types of laboratory data.

	Confidence Interpreting Information					
Data Type	Confident	Somewhat Confident	Not Confident	Don't know what this is	Total Confident*	Total Response
MIRU-VNTR	64.7%	29.4%	5.9%	0.0%	94.1%	100.0%
RFLP	29.4%	5.9%	35.3%	29.4%	35.3%	100.0%
Spoligotyping	23.5%	11.8%	23.5%	41.2%	35.3%	100.0%
Phenotypic DST	58.8%	23.5%	11.8%	5.9%	82.3%	100.0%
Molecular DST	58.8%	23.5%	11.8%	5.9%	82.3%	100.0%
SNPs conferring drug resistance	41.2%	29.4%	23.5%	5.9%	70.6%	100.0%
Genomic clusters	52.9%	29.4%	11.8%	5.9%	82.3%	100.0%
SNPs (mutations)	47.1%	35.2%	11.8%	5.9%	82.3%	100.0%
SNP distance between isolates	35.3%	41.2%	17.6%	5.9%	76.5%	100.0%
Phylogenetic tree	35.4%	29.4%	17.6%	17.6%	64.8%	100.0%
Percentage of genome covered	29.4%	29.4%	35.3%	5.9%	58.8%	100.0%
Genome sequencing quality metrics	29.4%	29.4%	29.4%	11.8%	58.8%	100.0%
Number of reads mapped	29.4%	29.4%	29.4%	11.8%	58.8%	100.0%
Depth of sequencing coverage	29.4%	29.4%	29.4%	11.8%	58.8%	100.0%

*Sum of confident and somewhat confident responses

Table S4. Task and Data Questionnaire respondents' confidence in the ability of genomic data to perform various laboratory tasks.

Task	Task Type	Level of Confidence			
		It can do this	It may be able to do this	It can't do this	Don't know what this is
Organism speciation	Diagnosis	76.5%	17.9%	5.4%	0.0%
Diagnose active TB		29.4%	23.5%	47.1%	0.0%
Predict drug susceptibility	Treatment	52.9%	47.1%	0.0%	0.0%
Inform choice of therapy		35.3%	64.7%	0.0%	0.0%
Monitor treatment progress		5.9%	47.1%	41.2%	5.9%
Identify epidemiologically related patients	Surveillance	58.8%	41.2%	0.0%	0.0%
Identify transmission events		41.2%	52.9%	5.9%	0.0%
Rule out transmission events		64.7%	29.4%	5.9%	0.0%
Assign patient to existing TB cluster		70.0%	29.4%	0.0%	0.0%

Table S5. Task and Data Questionnaire respondents' identification of laboratory-associated barriers impacting their workflows.

	Diagnosis	Treatment	Surveillance*
	Respondents = 6		Respondents = 5
No issues	0 (0.0%)	0 (0.0%)	NA
Need for additional data	0 (0.0%)	2 (33.3%)	3 (60.0%)
Timeliness of results	5 (83.3%)	5 (83.3%)	NA
Results provided over multiple unconnected documents	5 (83.3%)	5 (83.3%)	NA
Difficultly interpreting lab results	2 (33.3%)	3 (50.0%)	4 (80.0%)
Lab data is not routinely provided	0 (0.0%)	1 (16.7%)	3 (60.0%)
Lab data is not linked to patient data	1 (16.7%)	3 (50.0%)	1 (20.0%)
Other	2 (33.3%)	1 (16.7%)	NA

*Question only asked of respondents reporting a role involving TB surveillance.

Other responses provided as free text included:

- Need immediate testing for second-line drugs
- Need mutation details to get proxy for resistance while awaiting phenotypic DST results
- Need strain details to investigate transmission dynamics
- Need details on unusual cases/clusters
- Patient data must be manually entered

Table S6. Summary of questions asked in the Design Choice Questionnaire, including preferred response.

Question	Options	Participant Preference	Classification	Question Type
1 to 4	NA	NA	Demographic	NA
5	A - With bolding<br>B - Without bolding<br>C - They are equally informative	A - With Bolding	Design	Multiple Choice
6	A - Speciation<br>B - Organism (Control)<br>C - Diagnosis<br>D - Species	B - Organism (Control)	Wording	Rank
7	A - Full Sentence<br>B - Summary	A - Full Sentence	Wording	Rank
8	A - Drug Resistance (Control)<br>B - Drug Sensitivity<br>C - Drug Susceptibility<br>D - Treatment	C - Drug Susceptibility	Wording	Rank
9	A - 3 letter abbreviation (e.g. INH) (Control)<br>B - Full name (e.g. Isoniazid)<br>C - Show me everything (e.g. Isonizaid (INH,H))<br>D - They are equally informative	B - Full Name	Wording	Multiple Choice
10	A - 1 letter abbreviation (e.g. S,R,U) (Control)<br>B - Full text (e.g. Susceptibile, Resistant, Unknown)<br>C - They are equally informative	B - Full Name	Wording	Multiple Choice
11A	A - No, I am not interested in mutation data<br>B - Yes, on the same table with drug susceptibility data (Control)<br>C - Yes, but on the other side of the report	C - Yes, but on the other side of the report	Design	Multiple Choice
11B	A - Gene abbreviation<br>B - Base pair change<br>C - Amino acid change<br>D - # of reads at that position<br>E - # of reads supporting the mutation	A - Gene abbreviation	Design	Multiple Choice

12	A - Basic (Control)<br>B - Alert glyphs<br>C - Shaded<br>D - Bolded	D - Shaded	Design	Rank
13	A - Basic (Control)<br>B - Summary sentence<br>C - Tick boxes	C - Tick boxes	Design	Rank
14	A - Relatedness (Control)<br>B - Epidemiology<br>C - Cluster Detection	C - Cluster Detection	Wording	Rank
15	A - Percent Match (Control)<br>B - Organism Name	B - Organism Name	Design	Multiple Choice
16	A - Drugs listed by category<br>B - Prediction by drug<br>C - Summary sentence<br>D - Drugs listed by category bin<br>E - Abbreviated prediction by drug (Control)	A - Drugs listed by category<br>B - Prediction by drug	Design	Rank
17	A - # of cases with spark line<br>B - # of isolates related table<br>C - Table + graph of isolates by SNP distance<br>D - Table + phylogenetic tree<br>E - Related isolates with SNP difference details<br>F - Summary with related isolates per year	D - Table + Phylogenetic Tree	Design	Rank
18	A - Summary statement<br>B - No summary statement	A - Summary Statement	Design	Rank
19	A - One column<br>B - Two column	B - Two column	Design	Rank
21 to 23	NA	NA	Full Report	Likert
24	A - Dark heading<br>B - Gray heading<br>C - Light heading<br>D - Pictures		Full Report	Rank

Figure S1. Survey responses with confidence intervals. Panel A: Wording choices. Panel B: Design choices. Panel C: Full reports.

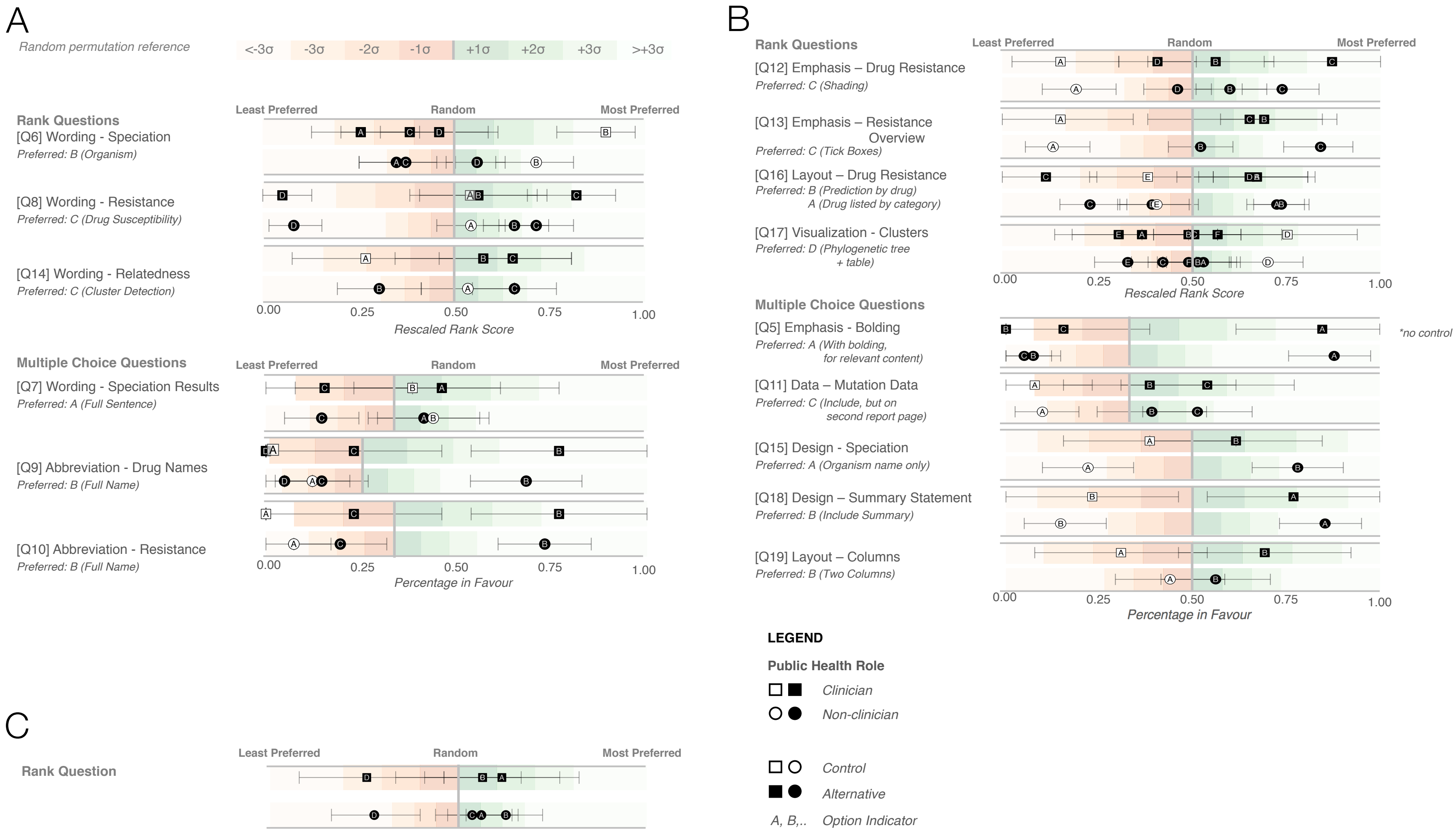

THE UNIVERSITY OF BRITISH COLUMBIA

COMPASS-TB Report Design Questionnaire

Page 1

Description and Consent

Many public health agencies are starting to use whole genome sequencing (reading every letter of an organism's DNA) as a tool for diagnosing infections, predicting what antibiotics an organism is sensitive or resistant to, and identifying closely related isolates that might suggest an outbreak. Last year, a study in The Lancet Infectious Diseases showed that when this technique is used in the tuberculosis laboratory, we can generate all the usual results that one has come to expect from a reference mycobacteriology lab, but we can do so much faster and at lower cost. As a result of this study, groups like Public Health England, the BC Centre for Disease Control, and the US Centers for Disease Control and Prevention are all using genomics to analyze their incoming mycobacterial isolates.

Sequencing a bacterial genome generates a lot of information, only some of which might be needed to manage a patient's infection. We are interested in designing a new lab report form that will help to communicate tuberculosis genomic data in a clear, concise, and meaningful way that will help those in the tuberculosis community - clinicians, epidemiologists, laboratory scientists, and more - in their daily work. There is a large field of research into how to present data in a way that makes it easily interpretable - we will be using principles from this field in designing our new report format, which will be shared with public health laboratories so that they may choose to use it in their own reporting.

By participating in this survey, you will help us better understand how you use lab data in your daily tuberculosis-related work. The answers from this survey will help us to design a series of sample reports, which we will test later in the year through a second survey.

Today's survey is divided into several parts. We'd like everyone to complete Parts I and II, which ask questions about your job and your familiarity with concepts and data types. Part III, on tasks related to diagnosis and treatment, will only be asked to physicians/clinicians. Part IV, on contact tracing and outbreak management, will be asked of all participants. Part V, on surveillance, will only be asked of epidemiologists, surveillance analysts, and researchers. All participants will be asked for (optional) email contact information in Part VI.

Consent for Participation

STUDY PROCEDURES:

If you agree to voluntarily participate in this research, your participation will include the following online survey (estimated completion time 15-30 minutes) in which you will be asked questions about how you use TB laboratory data in your work. At the end of the survey, you may choose to provide an email address if you'd like to be entered into a draw for an Apple Store gift card, or receive the final results of the study.

There are no known or anticipated risks to you by participating in this research. An optional benefit is receiving the results of the study via an emailed report at the project's conclusion, which will include a template for the final report design that participants may use in their own work. Study results will be also shared with the research community through open-access publications, conference reports, tweets and other social media postings.

MEASURES TO MAINTAIN CONFIDENTIALITY

Data from this study will be coded anonymously: a unique anonymous identifier will be used in place of the optional email addresses, which will be saved separately for the purposes of the gift card draw and sending information about the final report to participants. After analysis, the anonymized data will be saved in electronic format and made publicly available online for use by the research community.

CONTACTS FOR COMPLAINTS OR CONCERNS

Geoff McKee is a resident physician in Public Health and Preventive Medicine at the University of British Columbia and you may contact him if you have any further questions by email at gwmckee@alumni.ubc.ca or by phone at 250-818-3448.

If you have any concerns or complaints about your rights as a research participant and/or your experiences while participating in this study, contact the Research Participant Complaint Line in the UBC Office of Research Ethics at 604-822-8598 or if long distance e-mail RSIL@ors.ubc.ca or call toll free 1-877-822-8598.

Taking part in this study is entirely up to you. You have the right to refuse to participate in this study. If you decide to take part, you may choose to pull out of the study at any time without giving a reason.

By completing the questionnaire, you are consenting to participate in this research.

PRINCIPAL INVESTIGATOR:
Jennifer Gardy, School of Population & Public Health, Tel. 604-707-2488

CO-INVESTIGATORS:
Geoff McKee, School of Population and Public Health, Tel. 250-818-3448
Anamaria Crisan, School of Population and Public Health, Tel. 604-707-2510
Tamara Munzner, Department of Computer Science, Tel. 604- 827-5200

SPONSORS:
BCCDC Foundation for Population & Public Health
Genome British Columbia

UBC RISE NUMBER: H10-03336

☐ I Agree

PART I – OCCUPATION AND SUBJECT AREA KNOWLEDGE QUESTIONS

All participants are asked to complete this first part of the survey: we'd like to find out more about you, your background, and your general attitude towards genomics in public health.

1. What is your role in tuberculosis diagnosis, treatment, management, and/or surveillance? You may select more than one role.

[Select as many as apply]

- ☐ Clinical management - I work directly with TB patients, providing care and/or case management
- ☐ Laboratory work – I work in a mycobacteriology laboratory setting where I am involved with lab testing for TB
- ☐ Surveillance/epidemiology - I work with TB data to understand patterns in disease occurrence
- ☐ Research - I carry out academic research into TB
- ☐ Other, please specify... Type here

What is your clinical role?

[Select one option]

- ○ Physician/Clinician
- ○ Nurse
- ○ Other, please specify... Type here

2. Who is your primary employer?

[Select as many as apply]

- ☐ Public Health Organization - e.g. Public Health England, CDC
- ☐ Private Clinic/Primary Care - e.g. a doctor's office
- ☐ Hospital
- ☐ Academic Institution
- ☐ Other, please specify... Type here

3. In what country do you work?

[Select one option]

- ○ England
- ○ Canada
- ○ USA
- ○ Other, please specify... Type here

4. How many years of experience do you have working in the field of tuberculosis?

[Number of years]

Type here

5. Please indicate the highest level of training (if any) you have in the following subject areas:

* By professional experience, we mean collaborating with others on a project
** By continuing education, we mean attending workshops, training sessions, or self-directed learning

	None	Undergraduate	Graduate Masters, PhD, Medical Training	Professional Experience*	Continuing Education**
Molecular Biology or Biochemistry	☐	☐	☐	☐	☐
Epidemiology	☐	☐	☐	☐	☐
Biostatistics	☐	☐	☐	☐	☐
Bioinformatics	☐	☐	☐	☐	☐
Genomics	☐	☐	☐	☐	☐
Infectious Diseases	☐	☐	☐	☐	☐
Respiratory Medicine	☐	☐	☐	☐	☐

6. Have you ever heard of or been involved in a research project that used whole genome sequencing data to diagnose or characterize tuberculosis infections or understand tuberculosis epidemiology?

[Select one option]

- ○ Yes - I have heard about these sorts of studies but have not been involved in one
- ○ Yes - I have worked on one of these studies
- ○ No - I am not familiar with TB genomics studies

7. How enthusiastic are you about public health agencies using genome sequencing to understand and diagnose infectious diseases?

[Select one option]

- ○ Very enthusiastic – we should be using genomics now
- ○ Enthusiastic – genomics has a lot of potential, but still needs to be validated for clinical use
- ○ Neutral - I don't have a strong opinion on genomics in public health
- ○ Skeptical – genomics may be useful, but there is no clear application
- ○ It's all hype – genomics hasn't proven itself to be more useful than the techniques we currently use

PART II – FAMILIARITY WITH DATA TYPES

All participants are asked to complete this second part of the survey: we'd like to hear about the many types of TB laboratory data you might encounter in your work.

8. How frequently do you foresee yourself using the following data types in your future, routine work?

[Select one option per data type]

	Never	Rarely	Sometimes	Often	All the time	I Don't Know What This Is
Patient identifiers (Name, age, location)	○	○	○	○	○	○
Patient's own prior tuberculosis test results	○	○	○	○	○	○
Requester identifiers (Name, contact, copy to etc.)	○	○	○	○	○	○
Reviewer identifiers (Name, position etc.)	○	○	○	○	○	○
Type of sample (Sputum, fine needle aspirate etc)	○	○	○	○	○	○
Sample collection site (lymph node, peripheral blood draw etc.)	○	○	○	○	○	○
Sample collection date	○	○	○	○	○	○
Interpretation or comments from reviewer	○	○	○	○	○	○
Tuberculin Skin Test Results	○	○	○	○	○	○
Interferon Gamma Release Assay (IGRA) results	○	○	○	○	○	○
Chest X-ray results	○	○	○	○	○	○
Acid Fast Bacilli (AFB) Smear results	○	○	○	○	○	○
Culture results	○	○	○	○	○	○
Speciation (M. tuberculosis, MAC, M. bovis etc.)	○	○	○	○	○	○
Phenotypic drug susceptibility testing - determined by culture	○	○	○	○	○	○
Molecular drug susceptibility testing - determined by PCR or LIne Probe Assay (LPA)	○	○	○	○	○	○
Specific mutations conferring drug resistance (Resistotype)	○	○	○	○	○	○
Spoligotype	○	○	○	○	○	○
MIRU-VNTR	○	○	○	○	○	○
Restriction fragment length polymorphisms (RFLP)	○	○	○	○	○	○
Cluster Assignment	○	○	○	○	○	○
Single Nucleotide Polymorphism/Variant distance from other isolates	○	○	○	○	○	○
Phylogenetic Tree	○	○	○	○	○	○

	Never	Rarely	Sometimes	Often	All the time	I Don't Know What This Is
Laboratory performance measures (Sequence quality, coverage etc.)						

9. How would you describe your ability to interpret the following data?

To help you choose your answers, we suggest the following scheme:

- *Don't know what it is*: you are unaware of this data type
- *Not confident:* you know what these data are, but you are not certain how to interpret the data for clinical management, surveillance, or research.
- *Somewhat confident:* you know what these data are and are capable of interpreting it, but you usually seek out a confirmation for your interpretation
- *Confident:* you understand how to interpret this data and are confident in using it in your practice

	Don't know what this is	Not Confident	Somewhat Confident	Confident
Spoligotyping				
RFLP				
MIRU-VNTR				
Single Nucleotide Polymorphisms (mutations)				
Phenotypic Drug Susceptibility Testing from culture				
Molecular Drug Susceptibility Testing from PCR or LPA				
Single nucleotide polymorphisms/variants (mutations) conferring drug resistance				
Phylogenetic Tree				
Genetic distance between cases measured in Single Nucleotide Polymorphisms/Variants (mutations)				
Genomic Clusters				
Genome sequencing quality metrics				
Number of reads mapped/unmapped				
Percentage of Genome Covered				
Depth of sequencing coverage				

10. How confident are you that genomic data can be used to correctly perform the following tasks?

	Don't know what this is	It can't do this	It may be able to do this	It can do this
Organism Speciation				
Diagnose active tuberculosis				
Predict Drug Susceptibility				
Inform a physician's choice of a therapeutic regimen				
Monitor treatment progress				
Identify epidemiologically related patients				

	Don’t know what this is	It can’t do this	It may be able to do this	It can do this
Identify transmission events	○	○	○	○
Rule out transmission events	○	○	○	○
Assign patient to existing tuberculosis cluster	○	○	○	○

PART III – TASKS RELATED TO DIAGNOSIS & TREATMENT

Only physicians/clinicians are asked to complete this part: our initial assessment indicated that only clinicians are involved in diagnosis and treatment, these questions should not be answered by nurses, researchers, epidemiologists, or biostatisticians as they are not directly involved in diagnosis and treatment.

11. Are you involved in the diagnosis and treatment of tuberculosis?

Yes No

12. What types of samples do you requisition or send to the laboratory?

[Select as many as apply]

- Sputum
- Bronchoscopy Wash
- Fine Needle Aspirate
- Biopsy
- Urine
- Other, please specify... Type here

13. Do you want to know any laboratory or bioinformatics quality metrics that may be associated with that data being reported to you?

[Select one option]

- Yes – I want to always want to have data quality metrics
- No – Data quality results are not relevant, the lab would not release low quality data and I trust their processes
- I don't know
- Other, please specify... Type here

14. In what format do you currently receive this data?

[Select as many as apply]

- Physical report mailed or faxed to me (hard copy)
- PDF report in electronic health record system (soft copy)
- Extracted data in electronic health record system (soft copy)
- Other, please specify... Type here

15. In the following question you will be provided with several clinical tasks in the form of narratives and be asked what data you would use to complete the task.

A. [Diagnose Latent Tuberculosis] You receive a laboratory report for a patient screened for tuberculosis who recently immigrated from India. Which of the following data types would you use / be required to make a diagnosis of latent tuberculosis?

B. [Diagnose Active Tuberculosis] You receive a laboratory report for a patient recently hospitalized with respiratory and constitutional symptoms suggestive of tuberculosis. Which of the following data types would you use / be required to make a diagnosis of active tuberculosis?

C. [Reactivation vs. New Acquisition] You receive a laboratory report for a patient confirming active tuberculosis. Which of the following data types would you use / be

required to differentiate between reactivation and new acquisition of tuberculosis?

D. [Characterize Transmission Risk] You have just diagnosed a patient with active tuberculosis and are determining what steps are necessary to prevent transmission to others. What data would you use / be required to characterize the patient's risk of transmission?

[Select as many as apply]

	A. Diagnose Latent Tuberculosis	B. Diagnose Active Tuberculosis	C. Reactivation vs. New Acquisition	D. Characterize Transmission Risk
Patient identifiers (Name, age, location)	☐	☐	☐	☐
Patient's own prior tuberculosis test results	☐	☐	☐	☐
Requester identifiers (Name, contact, copy to etc.)	☐	☐	☐	☐
Reviewer identifiers (Name, position etc.)	☐	☐	☐	☐
Type of sample (Sputum, fine needle aspirate etc)	☐	☐	☐	☐
Sample collection site (lymph node, peripheral blood draw etc.)	☐	☐	☐	☐
Sample collection date	☐	☐	☐	☐
Report release date	☐	☐	☐	☐
Interpretation or comments from reviewer	☐	☐	☐	☐
Tuberculin Skin Test Results	☐	☐	☐	☐
Interferon Gamma Release Assay (IGRA) results	☐	☐	☐	☐
Chest X-ray results	☐	☐	☐	☐
Acid Fast Bacilli Smear results	☐	☐	☐	☐
Culture results	☐	☐	☐	☐
Speciation (m. tuberculosis, MAC, m. bovis etc.)	☐	☐	☐	☐
Phenotypic drug susceptibility testing	☐	☐	☐	☐
Predicted (in silico) drug susceptibility testing	☐	☐	☐	☐
Specific Mutations conferring drug resistance (Resistotype)	☐	☐	☐	☐
Spoligotype	☐	☐	☐	☐
MIRU-VNTR	☐	☐	☐	☐
Restriction fragment length polymorphisms (RFLP)	☐	☐	☐	☐
Cluster assignment	☐	☐	☐	☐
Single Nucleotide Polymorphism/Variant distance from other isolates	☐	☐	☐	☐
Phylogenetic tree	☐	☐	☐	☐
Laboratory performance measures (Sequence quality, coverage etc.)	☐	☐	☐	☐

16. When you are using laboratory data to diagnose a patient with active TB, you encounter the following challenges:

[Select as many as apply]

- ☐ No challenges - the lab data I currently receive is sufficient
- ☐ The lab data I currently receive does not help me to make a diagnosis
- ☐ I would like to receive data faster to make a more timely diagnosis
- ☐ Important results come at different times and/or in different documents
- ☐ I find it difficult to interpret the lab results I receive
- ☐ I am not regular receiving data that would help me to make a diagnosis
- ☐ The lab data I receive is not routinely linked to patient data
- ☐

Other, please specify... [Type here]

17. In the following question you will be provided with several clinical tasks in the form of narratives and be asked what data you would use to complete the task.

A. *[Choose Medications]* You are managing a patient who has just been diagnosed with active tuberculosis. What data would you use / be required to decide what medications should be prescribed for the patient?

B. *[Choose Duration of Treatment]* You are managing a patient who has just been diagnosed with active tuberculosis. What data would be required to decide the duration of treatment for the patient?

C. *[Assess Responsiveness to Treatment]* You continue to follow the patient as they proceed with the therapeutic regimen for active tuberculosis. What data would be required to assess their responsiveness to treatment?

[Select as many as apply]

	A. Choose Medications	B. Choose Duration of Treatment	C. Assess Responsiveness to Treatment
Patient identifiers (Name, age, location)	☐	☐	☐
Patient's own prior tuberculosis test results	☐	☐	☐
Requester identifiers (Name, contact, copy to etc.)	☐	☐	☐
Reviewer identifiers (Name, position etc.)	☐	☐	☐
Type of sample (Sputum, fine needle aspirate etc)	☐	☐	☐
Sample collection site (lymph node, peripheral blood draw etc.)	☐	☐	☐
Sample collection date	☐	☐	☐
Report release date	☐	☐	☐
Interpretation or comments from reviewer	☐	☐	☐
Tuberculin Skin Test Results	☐	☐	☐
Interferon Gamma Release Assay (IGRA) results	☐	☐	☐
Chest X-ray results	☐	☐	☐
Acid Fast Bacilli Smear results	☐	☐	☐
Culture results	☐	☐	☐
Speciation (m. tuberculosis, MAC, m. bovis etc.)	☐	☐	☐
Phenotypic drug susceptibility testing	☐	☐	☐
Predicted (in silico) drug susceptibility testing	☐	☐	☐
Specific Mutations conferring drug resistance (Resistotype)	☐	☐	☐
Spoligotype	☐	☐	☐
MIRU-VNTR	☐	☐	☐
Restriction fragment length polymorphisms (RFLP)	☐	☐	☐
Cluster assignment	☐	☐	☐
Single Nucleotide Polymorphism/Variant distance from other isolates	☐	☐	☐
Phylogenetic tree	☐	☐	☐
Laboratory performance measures (Sequence quality, coverage etc.)	☐	☐	☐

18. What are the main barriers for improving the efficiency of active TB treatment through the use of molecular laboratory data?

[Select as many as apply]

- ☐ There aren't any barriers
- ☐ Additional laboratory data is needed
- ☐ Timeliness of results being provided (too slow)

- [ ] Results provided over multiple unconnected documents
- [ ] Difficulty interpreting lab results
- [ ] Lab data is not routinely provided
- [ ] Lab data is not routinely linked to patient data
- [ ] Other, please specify... Type here

19. Do you have any additional comments you wish to make on the use of genomic and molecular data for active TB diagnosis and treatment?

Type here

Page 5

PART IV – CONTACT TRACING AND OUTBREAK MANAGEMENT

All participants are asked to complete this part: Contact tracing and outbreak management are performed by nurses, clinicians, epidemiologists, and sometimes also researchers.

20. Are you involved in the epidemiological aspects of TB management, including contact tracing and/or managing outbreak?

Note that surveillance - collating data for regional or national-level efforts - is not included here. It will be covered in the next section.
[Select only one]

Yes No

21. During your epidemiological work, do you directly review original lab reports?

[Select only one]

Yes No

Do you get aggregate extracted data?

[Select only one]

Yes No

22. In the following question you will be provided with several clinical tasks in the form of narratives and be asked what data you would use to complete the task.

A. [Guide Contact Tracing] You have been tasked with tracing potential contacts of a patient recently diagnosed with active tuberculosis. Which of the following data types would be useful in guiding contact tracing?

B. [Report to Public Health] You are a clinician managing several new cases of active tuberculosis and are concerned that they may represent a cluster. What data would influence your decision to report your concerns to public health?

C. [Define a Cluster] You are investigating increased incidence of tuberculosis in a rural community. What laboratory data would be required to define a cluster of tuberculosis cases?

D. [Connect Case to Existing Cluster] Following the identification of a cluster, new cases have been reported in a nearby community. What data would be required to connect these new cases to the existing cluster?

E. [Guide Public Health Response] What data would assist in guiding the public health response to the newly identified cluster?

[Select as many as apply]

	A. Guide Contact Tracing	B. Report to Public Health	C. Define a Cluster	D. Connect Case to Existing Cluster	E. Guide Public Health Response
Patient identifiers (Name, age, location)					
Patient's own prior tuberculosis test results					
Requester identifiers (Name, contact, copy to etc.)					
Reviewer identifiers (Name, position etc.)					
Type of sample (Sputum, fine needle aspirate etc)					
Sample collection site (lymph node, peripheral blood draw etc.)					
Sample collection date					
Report release date					
Interpretation or comments from reviewer					
Tuberculin Skin Test Results					
Interferon Gamma Release Assay (IGRA) results					
Chest X-ray results					
Acid Fast Bacilli Smear results					
Culture results					
Speciation (m. tuberculosis, MAC, m. bovis etc.)					
Phenotypic drug susceptibility testing					
Predicted (in silico) drug susceptibility testing					
Specific Mutations conferring drug resistance (Resistotype)					
Spoligotype					
MIRU-VNTR					
Restriction fragment length polymorphisms (RFLP)					
Cluster assignment					
Single Nucleotide Polymorphism/Variant distance from other isolates					
Phylogenetic tree					
Laboratory performance measures (Sequence quality, coverage etc.)					

PART V – SURVEILLANCE

Only epidemiologists, surveillance analysts, and researchers are asked to complete this part of the survey.

23. Are you involved in tuberculosis surveillance?

Yes No

24. What data does your institution currently use as part of its surveillance practices?

[Select as many as apply]

- Patient identifiers (Name, age, location)
- Patient's own prior tuberculosis test results
- Requester identifiers (Name, contact, copy to etc.)
- Reviewer identifiers (Name, position etc.)
- Type of sample (Sputum, fine needle aspirate etc)
- Sample collection site (lymph node, peripheral blood draw etc.)
- Sample collection date
- Report release date
- Interpretation or comments from reviewer
- Tuberculin Skin Test Results
- Interferon Gamma Release Assay (IGRA) results
- Chest X-ray results
- Acid Fast Bacilli Smear results
- Culture results
- Speciation (m. tuberculosis, MAC, m. bovis etc.)
- Phenotypic drug susceptibility testing
- Predicted (in silico) drug susceptibility testing
- Specific Mutations conferring drug resistance (Resistotype)
- Spoligotype
- MIRU-VNTR
- Restriction fragment length polymorphisms (RFLP)
- Cluster assignment
- Single Nucleotide Polymorphism/Variant distance from other isolates
- Phylogenetic tree
- Laboratory performance measures (Sequence quality, coverage etc.)

25. Is your institution planning to use more genomic data in the future?

[Select only one]

- ○ Yes – we're looking into it right now
- ○ Not yet – but we'd like to incorporate genomic data in the future
- ○ No and we have no plans to do so in the near future

How do envision genomic data being part of future surveillance efforts?

Type here

26. What is the main barrier of using genomic data more routinely as part of surveillance?

[Select as many as apply]

- ☐ Data is not consistently accessible
- ☐ Data are not consistently linked to relative patient data
- ☐ It is not clear how this data is useful for surveillance
- ☐ It is not clear how to interpret this data for surveillance purposes
- ☐ Difficulty interpreting lab results
- ☐ Other, please specify... Type here

PART VI – CONTACT INFORMATION

All participants are asked to complete this part of the survey.

Would you like to provide an email address so that we can contact you for the post-survey gift card draw and/or later email with the results of this survey? This contact information will be removed when we anonymize the survey data before making it available to other researchers.

[Select as many as apply]

- ☐ Yes, please enter me into the gift card draw for participants who complete this survey
- ☐ Yes, please send me the final results of this study

Email Address:

Type here

COMPASS-TB Report Design: Second Survey

0%

DESCRIPTION AND CONSENT

Many public health agencies are starting to use whole genome sequencing (reading every letter of an organism's DNA) as a tool for diagnosing infections, predicting what antibiotics an organism is sensitive or resistant to, and identifying closely related isolates that might suggest an outbreak. Last year, a study in The Lancet Infectious Diseases showed that when this technique is used in the tuberculosis laboratory, we can generate all the usual results that one has come to expect from a reference mycobacteriology lab, but we can do so much faster and at lower cost. As a result of this study, groups like Public Health England, the BC Centre for Disease Control, and the US Centers for Disease Control and Prevention are all using genomics to analyze their incoming mycobacterial isolates.

Sequencing a bacterial genome generates a lot of information, only some of which might be needed to manage a patient's infection. We are interested in designing a new lab report form that will help to communicate tuberculosis genomic data in a clear, concise, and meaningful way that will help those in the tuberculosis community - clinicians, epidemiologists, laboratory scientists, and more - in their daily work. There is a large field of research into how to present data in a way that makes it easily interpretable - we will be using principles from this field in designing our new report format, which will be shared with public health laboratories so that they may choose to use it in their own reporting.

By participating in this survey, you will help us better understand how lab data should be represented and what design elements should be used in the final report. The results of this survey will be used to construct a final prototype report that will be tested in a third and final survey later this year.

Consent for Participation

STUDY PROCEDURES:

If you agree to voluntarily participate in this research, your participation will include the following online survey (estimated completion time 15-30 minutes) in which you will be asked to compare different visual representations of genomic data and choose your preferred design. At the end of of the survey, you may choose to provide an email address if you'd like to be entered into a draw for an Amazon gift card.

There are no known or anticipated risks to you by participating in this research, and the benefit is receiving the results of the study via an emailed report at the project's conclusion, which will include a template for the final report design that participants may use in their own own work. Study results will be shared with the research community through open-access publications, conference reports, tweets and other social media postings.

MEASURES TO MAINTAIN CONFIDENTIALITY

Data from this study will be coded anonymously.

CONTACTS FOR COMPLAINTS OR CONCERNS

Geoff McKee is a resident physician in Public Health and Preventive Medicine at the University of British Columbia and you may contact him if you have any further questions by email at gwmckee@alumni.ubc.ca or by phone at 250-818-3448.

If you have any concerns or complaints about your rights as a research participant and/or your experiences while participating in this study, contact the Research Participant Complaint Line in the UBC Office of Research Ethics at 604-822-8598 or if long distance e-mail RSIL@ors.ubc.ca or call toll free 1-877-822-8598.

Taking part in this study is entirely up to you. You have the right to refuse to participate in this study. If you decide to take part, you may choose to pull out of the study at any time without giving a reason.

By completing the questionnaire, you are consenting to participate in this research.

PRINCIPAL INVESTIGATOR:
Jennifer Gardy, School of Population & Public Health, Tel. 604-707-2488

CO-INVESTIGATORS:
Geoff McKee, School of Population and Public Health, Tel. 250-818-3448
Anamaria Crisan, School of Population and Public Health, Tel. 604-707-2510

Tamara Munzner, Department of Computer Science, Tel. 604- 827-5200

SPONSORS:
BCCDC Foundation for Population & Public Health
Genome British Columbia

UBC RISE NUMBER: H10-03336

◯ I Agree

Next

Administrator

Page 1

About UBC
Contact UBC
About the University
News
Events
Careers
Make a Gift
Search UBC.ca

UBC Campuses
Vancouver Campus
Okanagan Campus

UBC Sites
Robson Square
Great Northern Way
Faculty of Medicine Across BC
Asia Pacific Regional Office

COMPASS-TB Report Design: Second Survey

16%

PART I – DEMOGRAPHICS

First, we have a few short questions about your background.

1. Do you work with tuberculosis patients or the Mycobacterium tuberculosis bacterium at all?

[Select one option]

Yes No

1B. What is your role in tuberculosis diagnosis, treatment, management, and/or surveillance?

[Select as many as apply]

- [ ] Physician - I work directly with TB patients, providing care and/or case management
- [ ] Nurse - I work directly with TB patients, providing care and/or case management
- [ ] Laboratory work – I work in a mycobacteriology laboratory setting where I am involved with lab testing for TB
- [ ] Surveillance/epidemiology - I work with TB data to understand patterns in disease occurrence
- [ ] Research - I carry out academic research into TB and/or M. tuberculosis
- [ ] Other, please specify... Type here

2. Do you work in public health microbiology or microbial genomics, whether on TB or another pathogen?

[Select one option]

Yes No

2B. What is your role in public health microbiology or microbial genomics?

[Select as many as apply]

- [ ] Clinical – I am directly involved in patient care and/or case management
- [ ] Bioinformatics – I use computational tools to analyse genomic data from pathogens
- [ ] Laboratory work – I am involved in directly handling and/or testing specimens
- [ ] Surveillance/epidemiology – I work with data to understand patterns in disease occurrence
- [ ] Research – I carry out academic research in public health and/or microbial genomics
- [ ] Other, please specify... Type here

2C. What pathogens do you work on?

[Select as many as apply]

- ☐ Respiratory infections (e.g. influenza, pertussis)
- ☐ Enteric infections (e.g. Salmonella, E. coli)
- ☐ Vector-borne disease (e.g. malaria, Zika)
- ☐ Blood-borne disease (e.g. HIV, hepatitis)
- ☐ Other, please specify... Type here

3. Who is your primary employer?

[Select as many as apply]

- ☐ Public Health Organization - e.g. Public Health England, CDC
- ☐ Private Clinic/Primary Care - e.g. a doctor’s office
- ☐ Hospital
- ☐ Academic Institution
- ☐ Other, please specify... Type here

4. In what country do you work?

[Select one option]

- ○ United Kingdom
- ○ Canada
- ○ USA
- ○ Other, please specify... Type here

Back Next

Administrator

Page 2

a place of mind
THE UNIVERSITY OF BRITISH COLUMBIA

About UBC
Contact UBC
About the University
News
Events
Careers
Make a Gift
Search UBC.ca

UBC Campuses
Vancouver Campus
Okanagan Campus

UBC Sites
Robson Square
Great Northern Way
Faculty of Medicine Across BC
Asia Pacific Regional Office

COMPASS-TB Report Design: Second Survey

33%

PART II – Design Elements

Laboratory results are usually communicated to end-users like doctors or public health officials in the form of a brief one- or two-page report. There are many different styles of lab report, from simple text documents to colourful pictorial reports. We are interested in understanding what sort of design choices can make a TB genomic laboratory report easy for end-users to read and to act upon. The report will contain information on what mycobacterial species a patient is infected with, what antibiotics their TB infection is susceptible or resistant to, and whether or not their TB isolate is related to other isolates and might be part of an outbreak.

Throughout the rest of the survey, we will be showing you some designs that show these different data – speciation, resistance, and epidemiological relatedness – in different ways. We want to find out which designs you prefer, so that these design elements can be incorporated into a final report design later in our project.

First, we will look at small elements of the report design.

5A. You are reading a summary of a patient's lab test results. Which of the following summary statement formats is better at communicating the information you need to know to do your job?

A

Summary

The specimen is positive for ***Mycobacterium tuberculosis***. It is **resistant to isoniazid and rifampin.** It belongs to a cluster, suggesting **recent transmission**.

B

Summary

The specimen is positive for *Mycobacterium tuberculosis*. It is resistant to isoniazid and rifampin. It belongs to a cluster, suggesting recent transmission.

[Select one option]

- ◯ A (with bolding)
- ◯ B (without bolding)
- ◯ They are equally informative.

5B. Please explain your choice or provide feedback.

[Optional]

Type here

6A. One section of the report will describe which mycobacterial species a patient was diagnosed with. Which headline best describes this section of the report?

A

Speciation
The specimen is positive for ***Mycobacterium tuberculosis***.

B

Organism
The specimen is positive for ***Mycobacterium tuberculosis***.

C

Diagnosis
The specimen is positive for ***Mycobacterium tuberculosis***.

D

Species
The specimen is positive for ***Mycobacterium tuberculosis***.

[Please rank your choices]

A (Speciation)	1	1
B (Organism)	2	2
C (Diagnosis)	3	3
D (Species)	4	4

6B. Please explain your choice or provide feedback.

[Optional]

Type here

7A. Which wording best conveys tuberculosis speciation results?

A

Speciation
The specimen is positive for ***Mycobacterium tuberculosis***.

B

Speciation
Organism: *Mycobacterium tuberculosis*

[Select one option]

- A (Full sentence)
- B (Summary)

○ They are equally informative

7B. Please explain your choice or provide feedback.

[Optional]

Type here

8A. The presence of particular mutations in a TB genome can be used to predict whether a specimen is sensitive or resistant to specific antibiotics. Which headline best describes this section of the report?

A

Drug Resistance

Drug	Prediction
Isoniazid	Resistant
Rifampin	Resistant
Ethambutol	Sensitive
Pyrazinimide	Sensitive

C

Drug Susceptibility

Drug	Prediction
Isoniazid	Resistant
Rifampin	Resistant
Ethambutol	Sensitive
Pyrazinimide	Sensitive

B

Drug Sensitivity

Drug	Prediction
Isoniazid	Resistant
Rifampin	Resistant
Ethambutol	Sensitive
Pyrazinimide	Sensitive

D

Treatment

Drug	Prediction
Isoniazid	Resistant
Rifampin	Resistant
Ethambutol	Sensitive
Pyrazinimide	Sensitive

[Please rank your choices]

A (Drug Resistance)	1	1
B (Drug Sensitivity)	2	2
C (Drug Susceptibility)	3	3
D (Treatment)	4	4

8B. Please explain your choice or provide feedback.

[Optional]

Type here

9A. There are many ways to represent a TB drug's name, from a single letter to a full name. Which naming scheme is most useful on a report?

[Select one option]

- Full Name (Ex. isoniazid)
- 3-letter abbreviation (Ex. INH)
- 1-letter abbreviation (Ex. H)
- Show me everything - (Ex. Isoniazid (INH, H))
- They are equally informative

9B. Please explain your choice or provide feedback.

[Optional]

Type here

10A. A specimen can be described as susceptible to an antibiotic (high likelihood of clinical success), resistant to an antibiotic (low likelihood of clinical success), intermediate (clinical success uncertain), or unknown (not enough information to draw a conclusion). Which naming scheme is most useful on a report?

[Select one option]

- Full Name (Ex. Susceptible, Resistant, Unknown)
- 1-letter abbreviation (Ex. S, R, U)
- They are equally informative

10B. Please explain your choice or provide feedback.

[Optional]

Type here

11A. Drug resistance in TB is caused by point mutations – single base-pair changes that alter the normal function of a gene or the protein it encodes. If a resistance phenotype is predicted from genomic data, would you want to know the exact mutation that caused it?

[Select one option]

- ◯ Yes – on the same table with the drug susceptibility data
- ◯ Yes, but on the other side of the report
- ◯ No – I am not interested in the mutation data

11B. What types of information related to the point mutation would you want to see?

[Select as many as apply]

- ☐ Gene abbreviation (e.g. katG, inhA)
- ☐ Base pair change (e.g. A1562C)
- ☐ Amino acid change (e.g. S531T)
- ☐ Number of sequencing reads that position (e.g. 48x)
- ☐ Number of reads supporting the mutation/coverage (e.g 47/48)

12A. Here are four ways of showing a result in which a specimen is resistant to two drugs. Which one is easiest for you to interpret?

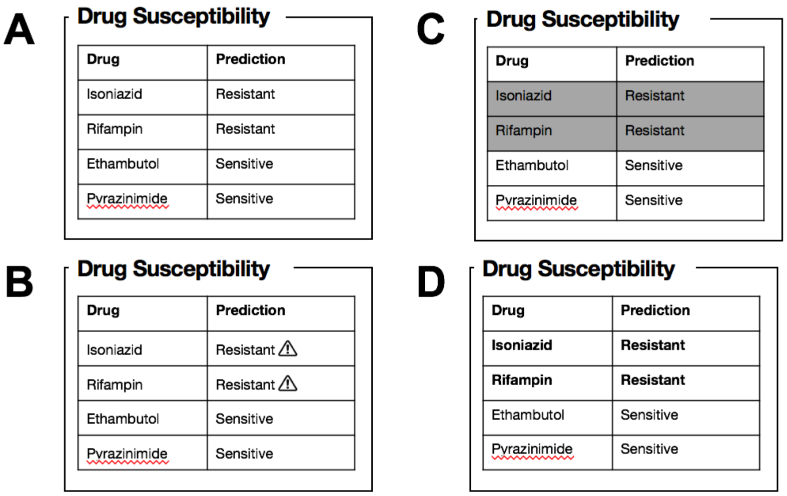

A — Drug Susceptibility

Drug	Prediction
Isoniazid	Resistant
Rifampin	Resistant
Ethambutol	Sensitive
Pyrazinimide	Sensitive

C — Drug Susceptibility

Drug	Prediction
Isoniazid	Resistant
Rifampin	Resistant
Ethambutol	Sensitive
Pyrazinimide	Sensitive

B — Drug Susceptibility

Drug	Prediction
Isoniazid	Resistant ⚠
Rifampin	Resistant ⚠
Ethambutol	Sensitive
Pyrazinimide	Sensitive

D — Drug Susceptibility

Drug	Prediction
**Isoniazid**	**Resistant**
**Rifampin**	**Resistant**
Ethambutol	Sensitive
Pyrazinimide	Sensitive

[Please rank your choices]

Choice	Rank	
A (Basic)	1	1
B (Alert Glyphs)	2	2
C (Shaded)	3	3
D (Bolded)	4	

4

12B. Please explain your choice or provide feedback.

[Optional]

Type here

13A. Depending on the resistance mutations observed, an isolate might be identified as having multidrug-resistant TB (MDR-TB). There are many ways this could be noted on the report.

A

Drug Susceptibility

Drug	Prediction
Isoniazid	Resistant
Rifampin	Resistant
Ethambutol	Sensitive
Pyrazinimide	Sensitive

B

Drug Susceptibility

Based on predicted antibiotic sensitivities, this individual has **multidrug-resistant (MDR) TB.**

Drug	Prediction
Isoniazid	Resistant
Rifampin	Resistant
Ethambutol	Sensitive
Pyrazinimide	Sensitive

C

Drug Susceptibility

- ☐ **Mono-resistant**
- ☑ **Multidrug-resistant (MDR)**
- ☐ **Extremely Drug Resistant (XDR)**

Drug	Prediction
Isoniazid	Resistant
Rifampin	Resistant
Ethambutol	Sensitive
Pyrazinimide	Sensitive

[Please rank your choices]

A (Basic)	1	1
B (Summary Sentence)	2	2
C (Tick Boxes)	3	3

13B. Please explain your choice or provide feedback.

[Optional]

Type here

14A. One section of the report will describe whether a patient's specimen is closely related to any specimens that were previously sequenced, suggesting the cases might be part of a cluster or outbreak. Which headline best describes this section of the report?

A

Relatedness

	**Likely Related** (less than 5 SNP Difference)	**Possibly Related** (6-30 SNP Differences)
**Number of isolates**	2	6

B

Epidemiology

	**Likely Related** (less than 5 SNP Difference)	**Possibly Related** (6-30 SNP Differences)
**Number of isolates**	2	6

C

Cluster Detection

	**Likely Related** (less than 5 SNP Difference)	**Possibly Related** (6-30 SNP Differences)
**Number of isolates**	2	6

[Please rank your choices]

A (Relatedness)	1	1
B (Epidemiology)	2	2
C (Cluster Detection)	3	3

14B. Please explain your choice or provide feedback.

[Optional]

Type here

Back Next

Administrator

Page 3

a place of mind
THE UNIVERSITY OF BRITISH COLUMBIA

About UBC
Contact UBC
About the University
News
Events
Careers
Make a Gift
Search UBC.ca

UBC Campuses
Vancouver Campus
Okanagan Campus

UBC Sites
Robson Square
Great Northern Way
Faculty of Medicine Across BC
Asia Pacific Regional Office

COMPASS-TB Report Design: Second Survey

50%

PART III – Report Sections

Now that we've looked at some individual design elements, we will next look at each of the three sections of the report: what organism is this, what antibiotics is it sensitive to, and is it related to other specimens. For each section, we will show you a few different representations of the same dataset; we want to know which one you prefer. Factors such as ease of readability, time taken to interpret the result, and aesthetics may all influence your choice

15A. Data on speciation and diagnosis is presented below in two different formats. Which do you find most interpretable?

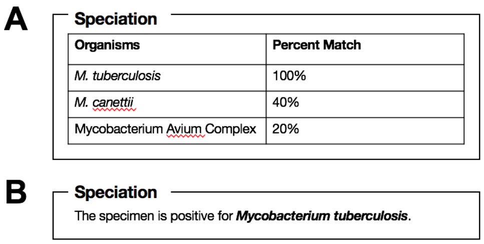

[Select one option]

- A (Percent match)
- B (Organism name)

15B. Please explain your choice or provide feedback.

[Optional]

Type here

16A. Data on drug susceptibility is presented below in a number of different formats. Which do you find most interpretable?

A

Drug Susceptibility

Prediction	Drugs
Sensitive	Ethambutol, Pyrazinimide
Resistant	Isoniazid, Rifampin
Indeterminate	-

B

Drug Susceptibility

Drug	Prediction
Isoniazid	Resistant
Rifampin	Resistant
Ethambutol	Sensitive
Pyrazinimide	Sensitive

C

Drug Susceptibility

The specimen is resistant to isoniazid, rifampin. It is sensitive to ethambutol and pyrazinamide.

D

Drug Susceptibility

SUSCEPTIBLE	RESISTANT	INDETERMINATE
Ethambutol<br>Pyrazinimide	Isoniazid<br>Rifampin	

E

Drug Susceptibility

INH	RIF	EMB	PZE
R	R	S	S

[Please rank your choices]

A (Drugs listed by category)	1	1
B (Prediction by drug)	2	2
C (Summary sentence)	3	3
D (Drugs listed by category bin)	4	4
E (Abbreviated prediction by drug)	5	5

16B. Please explain your choice or provide feedback.

[Optional]

Type here

17A. Data on relatedness to other isolates/clusters is presented below in a number of different formats. Which do you find most interpretable?

A

Relatedness

Similarity	SNP difference	Cluster trend (past 5 years)	#cases
Highly	0 to 5		10
Peripheral	6 to 12		25

B

Relatedness

	Likely Related (less than 5 SNP Difference)	Possibly Related (6-30 SNP Differences)
Number of isolates	2	6

C

Relatedness

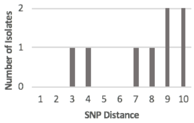

	Likely Related (< 5 SNPs)	Possibly Related (6-30 SNPs)
Number of isolates	2	6

D

Relatedness

	Likely Related (less than 5 SNP Difference)	Possibly Related (6-30 SNP Differences)
Number of isolates	2	6

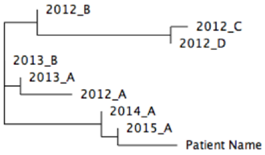

E

Relatedness

Isolate Name	SNP difference
2015_A	3
2014_A	4
2013_A	8
2013_B	7
2012_A	10
2012_B	9
2012_C	10
2012_D	9

F

Relatedness

This isolate belongs to a cluster of **8** genetically related cases, suggesting recent transmission.

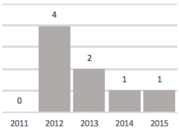

[Please rank your choices]

A (# of cases with spark line)	1	1
B (# of isolates related table)	2	2
C (Table + Graph of # of isolates by SNP distance)	3	3

D (Table + Phylogenetic Tree) 4 4

E (Related isolates with SNP difference details) 5 5

F (Summary with related isolates per year) 6 6

17B. Please explain your choice or provide feedback.

[Optional]

Type here

18. The reports below contrast between including a summary statement at the beginning of the report versus no summary. Please select which of the two potential layouts you find most preferable.

Click on images to zoom

A

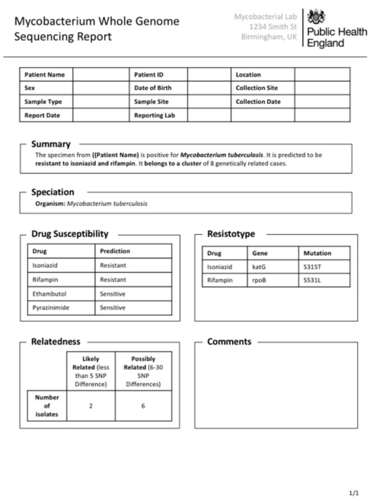

Mycobacterium Whole Genome Sequencing Report

Mycobacterial Lab
1234 Smith St
Birmingham, UK

Public Health England

**Patient Name**		**Patient ID**		**Location**	
**Sex**		**Date of Birth**		**Collection Site**	
**Sample Type**		**Sample Site**		**Collection Date**	
**Report Date**		**Reporting Lab**			

Summary

The specimen from **((Patient Name)** is positive for ***Mycobacterium tuberculosis***. It is predicted to be **resistant to isoniazid and rifampin**. It **belongs to a cluster** of 8 genetically related cases.

Speciation

Organism: *Mycobacterium tuberculosis*

Drug Susceptibility

**Drug**	**Prediction**
Isoniazid	Resistant
Rifampin	Resistant
Ethambutol	Sensitive
Pyrazinimide	Sensitive

Resistotype

**Drug**	**Gene**	**Mutation**
Isoniazid	katG	S315T
Rifampin	rpoB	S531L

Relatedness

	**Likely Related** (less than 5 SNP Difference)	**Possibly Related** (6-30 SNP Differences)
**Number of isolates**	2	6

Comments

1/1

B

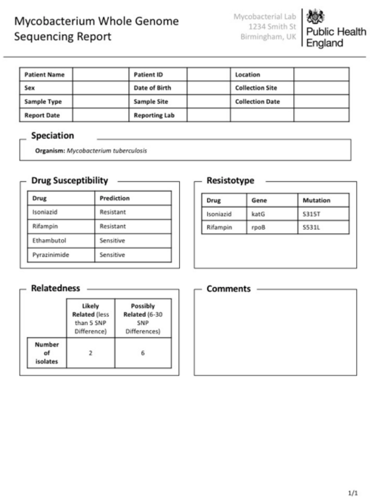

Mycobacterium Whole Genome Sequencing Report

Mycobacterial Lab
1234 Smith St
Birmingham, UK

Public Health England

**Patient Name**		**Patient ID**		**Location**	
**Sex**		**Date of Birth**		**Collection Site**	
**Sample Type**		**Sample Site**		**Collection Date**	
**Report Date**		**Reporting Lab**			

Speciation

Organism: *Mycobacterium tuberculosis*

Drug Susceptibility

**Drug**	**Prediction**
Isoniazid	Resistant
Rifampin	Resistant
Ethambutol	Sensitive
Pyrazinimide	Sensitive

Resistotype

**Drug**	**Gene**	**Mutation**
Isoniazid	katG	S315T
Rifampin	rpoB	S531L

Relatedness

	**Likely Related** (less than 5 SNP Difference)	**Possibly Related** (6-30 SNP Differences)
**Number of isolates**	2	6

Comments

1/1

[Select one option]

- A (Summary statement)
- B (No summary Statement)

19. The reports below show two potential ways to layout the speciation, drug susceptibility, and relatedness information – with categories presented in either one or two columns. Please select which of the two potential layouts you find most preferable.

Click on images to zoom

A

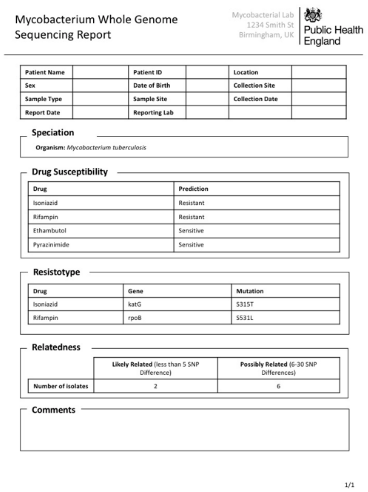
Mycobacterium Whole Genome Sequencing Report

Mycobacterial Lab
1234 Smith St
Birmingham, UK

Public Health England

Patient Name		Patient ID		Location	
Sex		Date of Birth		Collection Site	
Sample Type		Sample Site		Collection Date	
Report Date		Reporting Lab			

Speciation

Organism: *Mycobacterium tuberculosis*

Drug Susceptibility

Drug	Prediction
Isoniazid	Resistant
Rifampin	Resistant
Ethambutol	Sensitive
Pyrazinimide	Sensitive

Resistotype

Drug	Gene	Mutation
Isoniazid	katG	S315T
Rifampin	rpoB	S531L

Relatedness

	Likely Related (less than 5 SNP Difference)	Possibly Related (6-30 SNP Differences)
Number of isolates	2	6

Comments

1/1

B

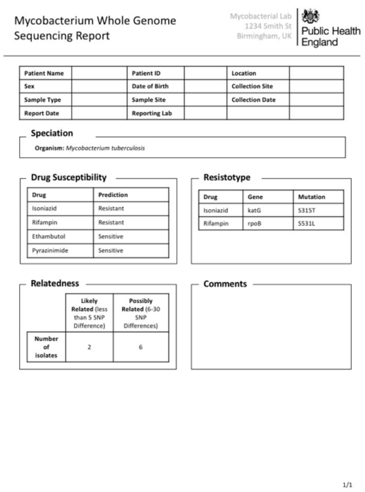
Mycobacterium Whole Genome Sequencing Report

Mycobacterial Lab
1234 Smith St
Birmingham, UK

Public Health England

Patient Name		Patient ID		Location	
Sex		Date of Birth		Collection Site	
Sample Type		Sample Site		Collection Date	
Report Date		Reporting Lab			

Speciation

Organism: *Mycobacterium tuberculosis*

Drug Susceptibility

Drug	Prediction
Isoniazid	Resistant
Rifampin	Resistant
Ethambutol	Sensitive
Pyrazinimide	Sensitive

Resistotype

Drug	Gene	Mutation
Isoniazid	katG	S315T
Rifampin	rpoB	S531L

Relatedness

	Likely Related (less than 5 SNP Difference)	Possibly Related (6-30 SNP Differences)
Number of isolates	2	6

Comments

1/1

[Select one option]

- ◯ A (One column)
- ◯ B (Two column)

Back Next

Administrator

Page 4

About UBC
Contact UBC
About the University
News
Events
Careers
Make a Gift
Search UBC.ca

UBC Campuses
Vancouver Campus
Okanagan Campus

UBC Sites
Robson Square
Great Northern Way
Faculty of Medicine Across BC
Asia Pacific Regional Office

COMPASS-TB Report Design: Second Survey

66%

PART IV – Report Feedback

In the last part of the survey, we will show you four potential prototype reports. You will have seen some of the elements already – things like speciation and resistance prediction – but you'll also see new information, such as a quality report describing the genome sequencing analysis. The reports have been organized such that the most critical information appears on page one, with expanded details on page two. Please read carefully through both pages before answering the questions.

20A. Please review the following report and select the response indicating your agreement with the corresponding statements.

Click on images to zoom

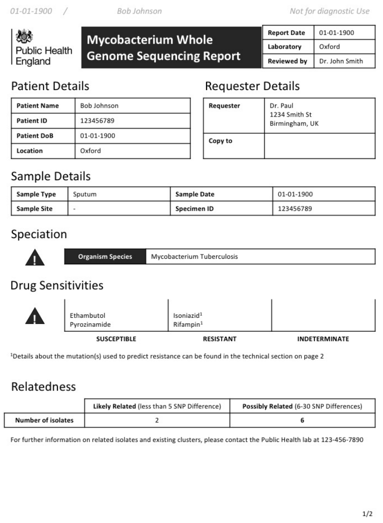

01-01-1900 / Bob Johnson Not for diagnostic Use

Public Health England

Mycobacterium Whole Genome Sequencing Report

**Report Date**	01-01-1900
**Laboratory**	Oxford
**Reviewed by**	Dr. John Smith

Patient Details

**Patient Name**	Bob Johnson
**Patient ID**	123456789
**Patient DoB**	01-01-1900
**Location**	Oxford

Requester Details

**Requester**	Dr. Paul<br>1234 Smith St<br>Birmingham, UK
**Copy to**	

Sample Details

**Sample Type**	Sputum	**Sample Date**	01-01-1900
**Sample Site**	-	**Specimen ID**	123456789

Speciation

**Organism Species**	Mycobacterium Tuberculosis

Drug Sensitivities

SUSCEPTIBLE	RESISTANT	INDETERMINATE
Ethambutol<br>Pyrozinamide	Isoniazid[1]<br>Rifampin[1]	

[1]Details about the mutation(s) used to predict resistance can be found in the technical section on page 2

Relatedness

	**Likely Related** (less than 5 SNP Difference)	**Possibly Related** (6-30 SNP Differences)
**Number of isolates**	2	6

For further information on related isolates and existing clusters, please contact the Public Health lab at 123-456-7890

1/2

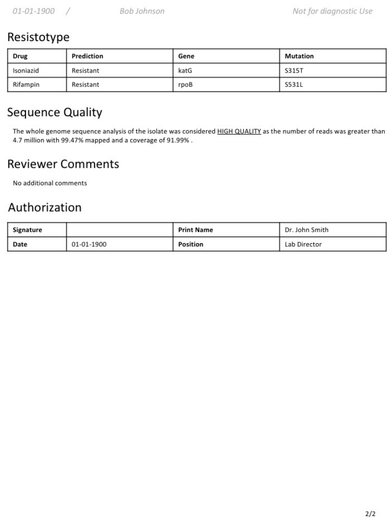

01-01-1900 / Bob Johnson Not for diagnostic Use

Resistotype

Drug	Prediction	Gene	Mutation
Isoniazid	Resistant	katG	S315T
Rifampin	Resistant	rpoB	S531L

Sequence Quality

The whole genome sequence analysis of the isolate was considered HIGH QUALITY as the number of reads was greater than 4.7 million with 99.47% mapped and a coverage of 91.99% .

Reviewer Comments

No additional comments

Authorization

**Signature**		**Print Name**	Dr. John Smith
**Date**	01-01-1900	**Position**	Lab Director

2/2

	Strongly Disagree	Disagree	Neutral	Agree	Strongly Agree
This report is easy to read.	○	○	○	○	○
I know what the information in this report means.	○	○	○	○	○
I can read this report and get the information I need quickly.	○	○	○	○	○
I feel that I can accurately interpret the information on this report.	○	○	○	○	○

20B. Please provide any additional comments you may have on the report.

[Optional]

Type here

21A. Please review the following report and select the response indicating your agreement with the corresponding statements.

Click on images to zoom

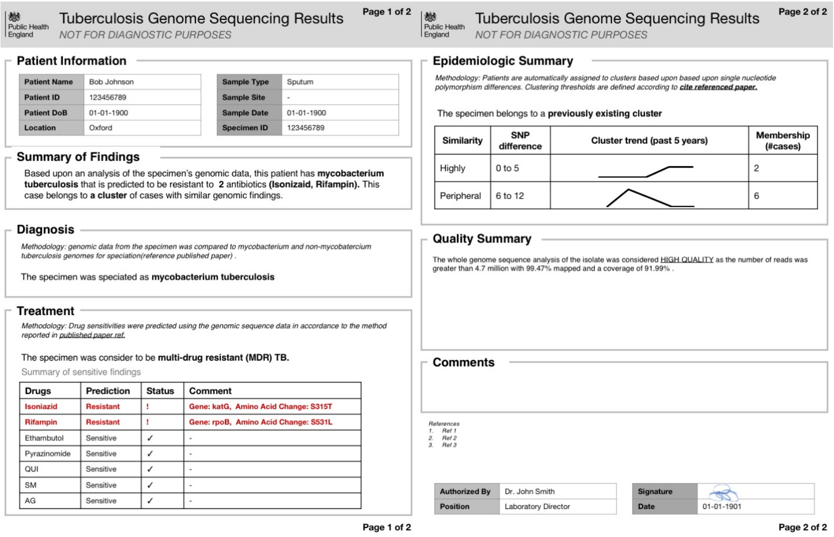

Public Health England

Tuberculosis Genome Sequencing Results — Page 1 of 2

NOT FOR DIAGNOSTIC PURPOSES

Patient Information

**Patient Name**	Bob Johnson	**Sample Type**	Sputum
**Patient ID**	123456789	**Sample Site**	-
**Patient DoB**	01-01-1900	**Sample Date**	01-01-1900
**Location**	Oxford	**Specimen ID**	123456789

Summary of Findings

Based upon an analysis of the specimen's genomic data, this patient has **mycobacterium tuberculosis** that is predicted to be resistant to **2** antibiotics **(Isonizaid, Rifampin).** This case belongs to **a cluster** of cases with similar genomic findings.

Diagnosis

Methodology: genomic data from the specimen was compared to mycobacterium and non-mycobatercium tuberculosis genomes for speciation(reference published paper) .

The specimen was speciated as **mycobacterium tuberculosis**

Treatment

Methodology: Drug sensitivities were predicted using the genomic sequence data in accordance to the method reported in published paper ref.

The specimen was consider to be **multi-drug resistant (MDR) TB.**

Summary of sensitive findings

Drugs	Prediction	Status	Comment
**Isoniazid**	**Resistant**	**!**	**Gene: katG, Amino Acid Change: S315T**
**Rifampin**	**Resistant**	**!**	**Gene: rpoB, Amino Acid Change: S531L**
Ethambutol	Sensitive	✓	-
Pyrazinomide	Sensitive	✓	-
QUI	Sensitive	✓	-
SM	Sensitive	✓	-
AG	Sensitive	✓	-

Page 1 of 2

Public Health England

Tuberculosis Genome Sequencing Results — Page 2 of 2

NOT FOR DIAGNOSTIC PURPOSES

Epidemiologic Summary

*Methodology: Patients are automatically assigned to clusters based upon based upon single nucleotide polymorphism differences. Clustering thresholds are defined according to **cite referenced paper.***

The specimen belongs to a **previously existing cluster**

Similarity	SNP difference	Cluster trend (past 5 years)	Membership (#cases)
Highly	0 to 5		2
Peripheral	6 to 12		6

Quality Summary

The whole genome sequence analysis of the isolate was considered HIGH QUALITY as the number of reads was greater than 4.7 million with 99.47% mapped and a coverage of 91.99% .

Comments

References
1. *Ref 1*
2. *Ref 2*
3. *Ref 3*

**Authorized By**	Dr. John Smith	**Signature**	
**Position**	Laboratory Director	**Date**	01-01-1901

Page 2 of 2

	Strongly Disagree	Disagree	Neutral	Agree	Strongly Agree
This report is easy to read.	○	○	○	○	○
I know what the information in this report means.	○	○	○	○	○
I can read this report and get the information I need quickly.	○	○	○	○	○
I feel that I can accurately interpret the information on this report.	○	○	○	○	○

21B. Please provide any additional comments you may have on the report.

[Optional]

Type here

22A. Please review the following report and select the response indicating your agreement with the corresponding statements.

Click on images to zoom

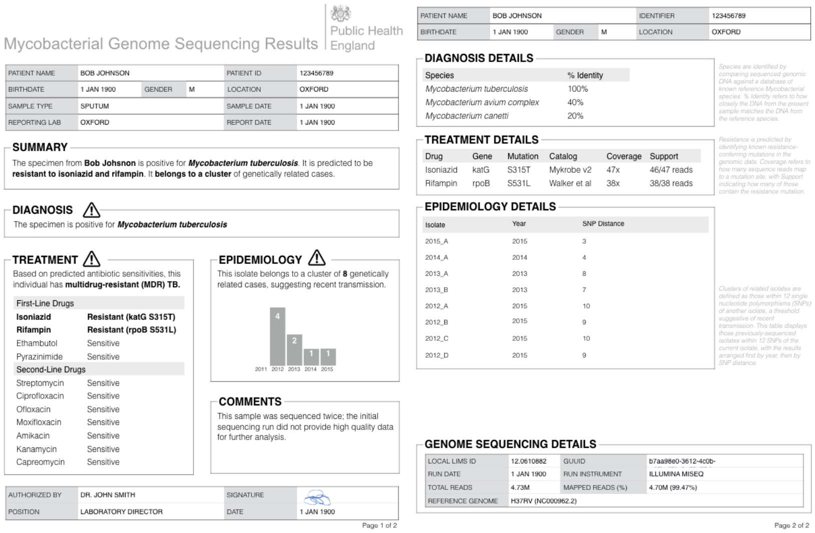

Mycobacterial Genome Sequencing Results | Public Health England

PATIENT NAME	BOB JOHNSON	PATIENT ID	123456789
BIRTHDATE	1 JAN 1900 GENDER M	LOCATION	OXFORD
SAMPLE TYPE	SPUTUM	SAMPLE DATE	1 JAN 1900
REPORTING LAB	OXFORD	REPORT DATE	1 JAN 1900

SUMMARY

The specimen from **Bob Johnson** is positive for ***Mycobacterium tuberculosis***. It is predicted to be **resistant to isoniazid and rifampin**. It **belongs to a cluster** of genetically related cases.

DIAGNOSIS ⚠

The specimen is positive for ***Mycobacterium tuberculosis***

TREATMENT ⚠

Based on predicted antibiotic sensitivities, this individual has **multidrug-resistant (MDR) TB.**

First-Line Drugs	
**Isoniazid**	**Resistant (katG S315T)**
**Rifampin**	**Resistant (rpoB S531L)**
Ethambutol	Sensitive
Pyrazinimide	Sensitive
**Second-Line Drugs**	
Streptomycin	Sensitive
Ciprofloxacin	Sensitive
Ofloxacin	Sensitive
Moxifloxacin	Sensitive
Amikacin	Sensitive
Kanamycin	Sensitive
Capreomycin	Sensitive

EPIDEMIOLOGY ⚠

This isolate belongs to a cluster of **8** genetically related cases, suggesting recent transmission.

COMMENTS

This sample was sequenced twice; the initial sequencing run did not provide high quality data for further analysis.

AUTHORIZED BY	DR. JOHN SMITH	SIGNATURE	
POSITION	LABORATORY DIRECTOR	DATE	1 JAN 1900

Page 1 of 2

PATIENT NAME	BOB JOHNSON	IDENTIFIER	123456789
BIRTHDATE	1 JAN 1900 GENDER M	LOCATION	OXFORD

DIAGNOSIS DETAILS

Species	% Identity
Mycobacterium tuberculosis	100%
Mycobacterium avium complex	40%
Mycobacterium canetti	20%

Species are identified by comparing sequenced genomic DNA against a database of known reference Mycobacterial species. % Identity refers to how closely the DNA from the present sample matches the DNA from the reference species.

TREATMENT DETAILS

Drug	Gene	Mutation	Catalog	Coverage	Support
Isoniazid	katG	S315T	Mykrobe v2	47x	46/47 reads
Rifampin	rpoB	S531L	Walker et al	38x	38/38 reads

Resistance is predicted by identifying known resistance-conferring mutations in the genomic data. Coverage refers to how many sequence reads map to a mutation site, with Support indicating how many of those contain the resistance mutation.

EPIDEMIOLOGY DETAILS

Isolate	Year	SNP Distance
2015_A	2015	3
2014_A	2014	4
2013_A	2013	8
2013_B	2013	7
2012_A	2015	10
2012_B	2015	9
2012_C	2015	10
2012_D	2015	9

Clusters of related isolates are defined as those within 12 single nucleotide polymorphisms (SNPs) of another isolate, a threshold suggestive of recent transmission. This table displays those previously-sequenced isolates within 12 SNPs of the current isolate, with the results arranged first by year, then by SNP distance.

GENOME SEQUENCING DETAILS

LOCAL LIMS ID	12.0610882	GUUID	b7aa98e0-3612-4c0b-
RUN DATE	1 JAN 1900	RUN INSTRUMENT	ILLUMINA MISEQ
TOTAL READS	4.73M	MAPPED READS (%)	4.70M (99.47%)
REFERENCE GENOME	H37RV (NC000962.2)		

Page 2 of 2

	Strongly Disagree	Disagree	Neutral	Agree	Strongly Agree
This report is easy to read.	○	○	○	○	○
I know what the information in this report means.	○	○	○	○	○
I can read this report and get the information I need quickly.	○	○	○	○	○
I feel that I can accurately interpret the information on this report.	○	○	○	○	○

22B. Please provide any additional comments you may have on the report.

[Optional]

Type here

23A. Please review the following report and select the response indicating your agreement with the corresponding statements.

Click on images to zoom

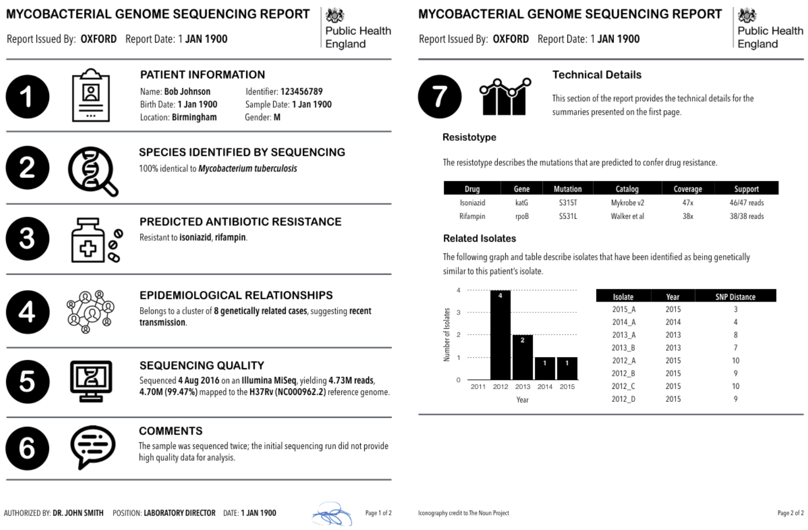
MYCOBACTERIAL GENOME SEQUENCING REPORT Public Health England

Report Issued By: **OXFORD** Report Date: 1 **JAN 1900**

1 **PATIENT INFORMATION**

Name: **Bob Johnson** Identifier: **123456789**
Birth Date: **1 Jan 1900** Sample Date: **1 Jan 1900**
Location: **Birmingham** Gender: **M**

2 **SPECIES IDENTIFIED BY SEQUENCING**

100% identical to ***Mycobacterium tuberculosis***

3 **PREDICTED ANTIBIOTIC RESISTANCE**

Resistant to **isoniazid**, **rifampin**.

4 **EPIDEMIOLOGICAL RELATIONSHIPS**

Belongs to a cluster of **8 genetically related cases**, suggesting **recent transmission**.

5 **SEQUENCING QUALITY**

Sequenced **4 Aug 2016** on an **Illumina MiSeq**, yielding **4.73M reads**, **4.70M (99.47%)** mapped to the **H37Rv (NC000962.2)** reference genome.

6 **COMMENTS**

The sample was sequenced twice; the initial sequencing run did not provide high quality data for analysis.

AUTHORIZED BY: **DR. JOHN SMITH** POSITION: **LABORATORY DIRECTOR** DATE: **1 JAN 1900** Page 1 of 2

MYCOBACTERIAL GENOME SEQUENCING REPORT Public Health England

Report Issued By: **OXFORD** Report Date: 1 **JAN 1900**

7 **Technical Details**

This section of the report provides the technical details for the summaries presented on the first page.

Resistotype

The resistotype describes the mutations that are predicted to confer drug resistance.

Drug	Gene	Mutation	Catalog	Coverage	Support
Isoniazid	katG	S315T	Mykrobe v2	47x	46/47 reads
Rifampin	rpoB	S531L	Walker et al	38x	38/38 reads

Related Isolates

The following graph and table describe isolates that have been identified as being genetically similar to this patient's isolate.

Isolate	Year	SNP Distance
2015_A	2015	3
2014_A	2014	4
2013_A	2013	8
2013_B	2013	7
2012_A	2015	10
2012_B	2015	9
2012_C	2015	10
2012_D	2015	9

Iconography credit to The Noun Project Page 2 of 2

	Strongly Disagree	Disagree	Neutral	Agree	Strongly Agree
This report is easy to read.	○	○	○	○	○
I know what the information in this report means.	○	○	○	○	○
I can read this report and get the information I need quickly.	○	○	○	○	○
I feel that I can accurately interpret the information on this report.	○	○	○	○	○

23B. Please provide any additional comments you may have on the report.

[Optional]

Type here

24A. The previous 4 report prototypes demonstrate different ways of presenting lab data from whole genome sequencing of a tuberculosis isolate. Which of the reports to you prefer?

Please see previous questions for enlarged images.

A

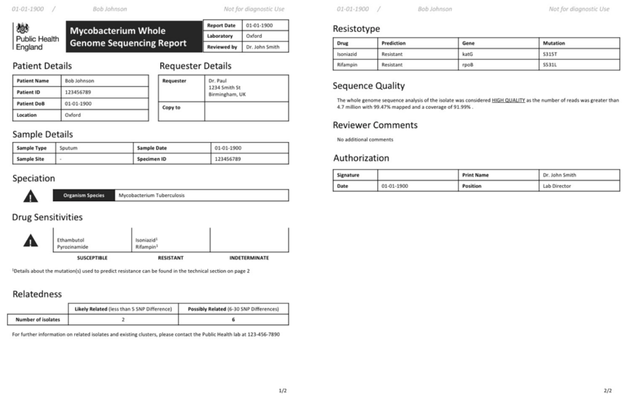
01-01-1900 / Bob Johnson Not for diagnostic Use

Public Health England

Mycobacterium Whole Genome Sequencing Report

**Report Date**	01-01-1900
**Laboratory**	Oxford
**Reviewed by**	Dr. John Smith

Patient Details

**Patient Name**	Bob Johnson
**Patient ID**	123456789
**Patient DoB**	01-01-1900
**Location**	Oxford

Requester Details

**Requester**	Dr. Paul<br>1234 Smith St<br>Birmingham, UK
**Copy to**	

Sample Details

**Sample Type**	Sputum	**Sample Date**	01-01-1900
**Sample Site**	-	**Specimen ID**	123456789

Speciation

**Organism Species**	Mycobacterium Tuberculosis

Drug Sensitivities

SUSCEPTIBLE	RESISTANT	INDETERMINATE
Ethambutol<br>Pyrozinamide	Isoniazid[1]<br>Rifampin[1]	

[1]Details about the mutation(s) used to predict resistance can be found in the technical section on page 2

Relatedness

	**Likely Related** (less than 5 SNP Difference)	**Possibly Related** (6-30 SNP Differences)
**Number of isolates**	2	6

For further information on related isolates and existing clusters, please contact the Public Health lab at 123-456-7890

1/2

01-01-1900 / Bob Johnson Not for diagnostic Use

Resistotype

Drug	Prediction	Gene	Mutation
Isoniazid	Resistant	katG	S315T
Rifampin	Resistant	rpoB	S531L

Sequence Quality

The whole genome sequence analysis of the isolate was considered HIGH QUALITY as the number of reads was greater than 4.7 million with 99.47% mapped and a coverage of 91.99% .

Reviewer Comments

No additional comments

Authorization

**Signature**		**Print Name**	Dr. John Smith
**Date**	01-01-1900	**Position**	Lab Director

2/2

B

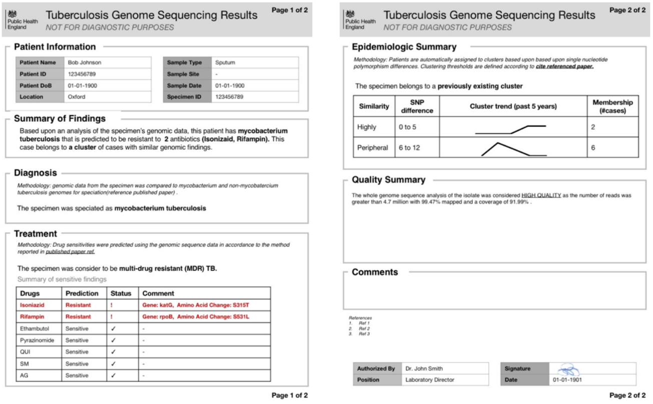

C

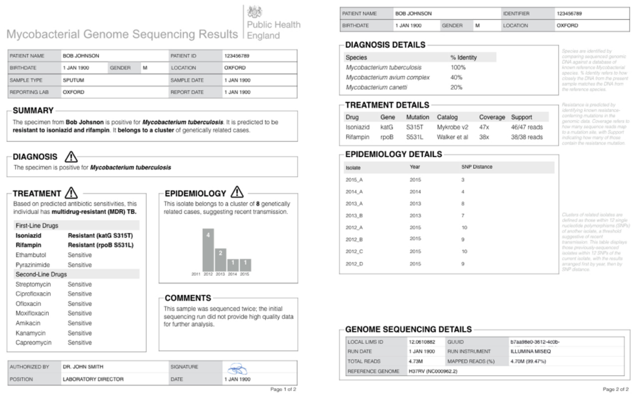

D

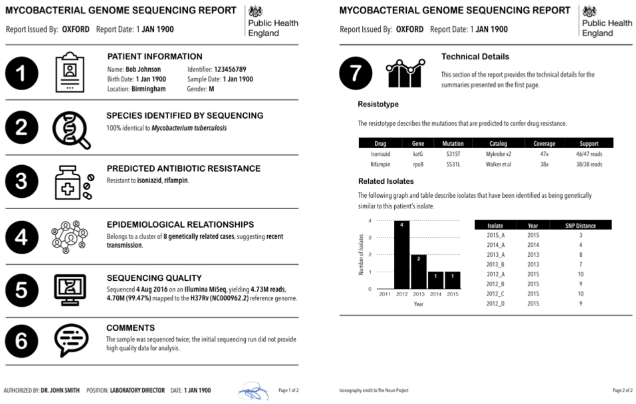

[Please rank your choices]

Choice	Rank
A (Dark heading)	1
B (Gray heading)	2
C (Light Heading)	3
D (Pictures)	4

24B. Please explain your choice or provide feedback.

[Optional]

Type here

Back Next

Administrator

Page 5

a place of mind
THE UNIVERSITY OF BRITISH COLUMBIA

About UBC
Contact UBC
About the University
News
Events
Careers
Make a Gift
Search UBC.ca

UBC Campuses
Vancouver Campus
Okanagan Campus

UBC Sites
Robson Square
Great Northern Way
Faculty of Medicine Across BC
Asia Pacific Regional Office

COMPASS-TB Report Design: Second Survey

83%

PART V – CONTACT INFORMATION

Thank you so much for taking part in our survey! Your responses will help us create a better, more interpretable laboratory report. You can follow our project's progress at Public Health InfoVis – we will be collating the results of this survey and releasing a summary report on the blog shortly. We are also happy to email you a copy of the report.

Don't forget, by having completed the survey, you are eligible to enter our draw for an Amazon gift card. To enter the draw, please enter an email address below.

25. Would you like to provide an email address so that we can contact you for the post-survey gift card draw and/or later email with the results of this survey?

This contact information will be removed when we anonymize the survey data before making it available to other researchers.

- [ ] Yes, please enter me into the gift card draw for participants who complete this survey
- [ ] Yes, please send me the final results of this study

Email Address:

Type here

Back Submit

Administrator

Page 6

About UBC
Contact UBC
About the University
News
Events
Careers
Make a Gift
Search UBC.ca

UBC Campuses
Vancouver Campus
Okanagan Campus

UBC Sites
Robson Square
Great Northern Way
Faculty of Medicine Across BC
Asia Pacific Regional Office

Final design walkthrough.

Shorthand for the different surveys / requirements documents

Abbreviation:	Examples:
EC: Expert Consults	EC-1 = Expert consult #1
S1: Survey 1 (task survey)	S1-Q10 = Survey 1 question 10
S2: Survey 2 (design survey)	S2-Q11A = Survey 2 question 11A
ISO: ISO15189 requirements document	S2-SR18 = Survey 2 survey respondent 18 (for text answers)

Justification for final design choices by section

1. **Summary Statement**
    a. On first page of report
    b. Summary sentence
    c. Bold important terms

2. **Organism**
    a. On first page of report
    b. Section title is Organism (supported by S2-Q6. 22/42 (52%) of respondents prefer "Organism" as top choice (32/42 preferred it as one of their top two choices). Next ranked was "Diagnosis" (12/42), but this was not preferred by clinicians; the choice was mainly driven by non-clinicians. Split preference for "Diagnosis" in all groups (12 would rank it best, 18 would rank it worst).
    c. Summary sentence with bolding to emphasize findings

3. **Drug Susceptibility:** in general, there was not a clear and obvious dislike of the control design (S2-Q16 "Abbreviated prediction by drug") because it was not consistently ranked as lowest preference, but it was not the most desirable choice for respondents. Clinicians tended to rank the control design as the lowest preference relative to non-clinicians.
    a. On first page of report
    b. Section title is Drug Susceptibility (supported by S2-Q8. Majority of respondents (20/42; 47.6%) preferred "Drug Susceptibility" as their first choice and 32/42 preferred it as one of their top two choices, but other options also selected (Drug Resistance, Drug Sensitivity). Anecdotal evidence that the title predicted drug resistance still controversial. Clinicians split nearly evenly between Drug Resistance & Drug Sensitivity (more so than non-clinicians), however Drug Susceptibility is preferred amongst top two choices. All groups agree "Treatment" is a bad title (35/42 respondents).
    c. Summary sentence to state in silico prediction (not phenotypic)
    d. Tick boxes to indicate mono, multi, or extensive drug resistance (supported by 30/42 who rated tick boxes as preferred choice, and majority rate basic (control report design) as least preferred (33/42). Good comment support for tick boxes too: S2-R5: "[..] Tick box is the most straightforward way [..] summary sentence [..]likely will be ignored"; S2-R23: "the less risk of misinterpretation of test data the better"
    e. Table listing predictions for drug susceptibility (supported by responses for S2-Q16. Many respondents felt that an organized table/bins would be the best, and when including the resistance information (section 5) the table was the easiest choice.)
        i. Categorize drugs by class
        ii. Categorize drugs by susceptible or resistant using full term (S2-Q16 top choices were to "list prediction by drug" (16/42) and also to "list prediction by category" (12/42). The design choices offered didn't quite do both, but the final design does. It categories drugs according to first and second line (not test on S2) and then by Sensitive / Resistant and finally lists each drug line by line.)
        iii. Full name (no abbreviation) for drugs
        iv. Highlight resistant drugs by shading (supported by S2-Q12 where majority preferred "shading" (25/42) over other options. Clear that basic (no emphasis on resistance) least preferred (30/42 ranked it last). Number of comments were made for showing resistance: S2-SR3 "report must call attention to drug resistance"; S2-R18 "MDR-TB should be flagged", S2-R11 "best highlights the MDR-TB", S2-SR16 "better to highlight what is working instead of what is not working", S2-SR24 "Bold gets confused with column headers")
        v. Indicate resistance prediction source (see 4. Resistance Information)

4. **Resistance Information**: majority of respondents do not care to see resistance information (S2-Q#; 26/42 info not wanted or on second page), but we chose to add it as short format addition to the resistance table as we had the space and included limited information.

a. Incorporated into Drug Susceptibility table
b. Column header: Resistance (Mutation)
c. Resistance indicated by Gene (Amino Acid Change) or "No mutation detected". (S2-Q11. 36/42 (85.7%) wanted gene abbreviation info included and more than 50% (24/42 in both cases) wanted to see amino acid change and number of reads covering the mutation. We decided to include only gene abbreviation and amino acid change. There are contradictory results for # of reads (24 want "coverage for reads supporting mutation", but only 10/42 want "# of sequencing reads at that position" – these are similar concepts, so odd that there isn't equivalent desire). Other data suggest clinicians in particular do want to see this kind of laboratory data (see 7. Laboratory Quality Data). Amino acid for people who wants to dive in deeper, but could be removed as gene was most relevant. S2-Q16 S2-SR20 "[cannot] a specimen is sensitive […] explain that the specimen does not harbor any known mutation associated to drug resistance.")

5. **Cluster Detection**: concerns raised about the relevance of this information at all: S2-SR18 "Cluster detection would only be fine for those who already know what a cluster is", S2-SR9 "Not sure what this conveys [..] What is the clinical action?"
    a. On second page of report
    b. Section title is Cluster Detection (supported by S2-Q14. All respondents ranked "cluster detection" as top choice (20/42) or top two choices (36/42), compared to 13/42 and 26/42 for control design ("Relatedness"). Also "cluster detection" or "epidemiology" was the most preferred by clinicians, while "relatedness" was the least preferred. In fact "Relatedness" seemed to be most preferred by non-clinician. Support also from comments: S2-SR23 "When I see this I think epidemiology and clusters; not relatedness", S2-SR11 "Cluster detection is important clinically and epidemiologically.")
    c. Table with phylogenetic tree (control option preferred)

6. **Laboratory Quality Data**
    a. Do not include laboratory (sample & sequence) QC data on report (Compared to the original report, this report does not have the laboratory technical details (i.e. percent mapping to reference, genome coverage, reference genome information etc.) because this was deemed not necessary information for any of the tasks that stakeholders (but especially clinicians) used to conduct their activities (S1). Including laboratory technical data considered harmful ("Why would the lab put out poor quality results for me to interpret?", "Isn't that up to the lab?" (EC)). This doesn't mean the data isn't collected and stored but that the data isn't presented on the clinical report. It can be moved to the second page of the report if necessary, but should not be featured on the front page.

ISO15189 Requirements

BSI Standards – BS EN ISO 15189:2012 Medical Laboratories- Requirements for quality and competence.

5.8 Reporting of results

5.8.1 General

- The results of each examination shall be reported accurately, clearly, unambiguously and in accordance with any specific instructions in the examination procedures.
- The laboratory shall define the format and medium of the report (i.e. electronic or paper) and the manner in which it is to be communicated from the laboratory.
- The laboratory shall have a procedure to ensure the correctness of transcription of laboratory results.
- Reports shall include the information necessary for the interpretation of the examination results.
- The laboratory shall have a process for notifying the requester when an examination is delayed that could compromise patient care.

5.8.2 Report attributes

- The laboratory shall ensure that the following report attributes effectively communicate laboratory results and meet the users' needs:
- comments on sample quality that might compromise examination results;
- comments regarding sample suitability with respect to acceptance/rejection criteria;
- critical results, where applicable;
- interpretive comments on results, where applicable, which may include the verification of the interpretation of automatically selected and reported results (see 5.9.1) in the final report.

5.8.3 Report content

- The report shall include, but not be limited to, the following:
    - a clear, unambiguous identification of the examination including, where appropriate, the examination procedure;
    - the identification of the laboratory that issued the report; Will this be Oxford or Birmingham?
    - identification of all examinations that have been performed by a referral laboratory;
    - patient identification and patient location on each page;
    - name or other unique identifier of the requester and the requester's contact details;
    - date of primary sample collection (and time, when available and relevant to patient care);
    - type of primary sample;
    - measurement procedure, where appropriate;
    - examination results reported in SI units, units traceable to SI units, or other applicable units;
    - biological reference intervals, clinical decision values, or diagrams/nomograms supporting clinical decision values, where applicable;
        - NOTE Under some circumstances, it might be appropriate to distribute lists or tables of biological reference intervals to all users of laboratory services at sites where reports are received.
    - interpretation of results, where appropriate;
        - NOTE Complete interpretation of results requires the context of clinical information that may not be available to the laboratory.
    - other comments such as cautionary or explanatory notes (e.g. quality or adequacy of the primary sample which may have compromised the result, results/interpretations from referral laboratories, use of developmental procedure
    - identification of examinations undertaken as part of a research or development programme and for which no specific claims on measurement performance are available;
    - identification of the person(s) reviewing the results and authorizing the release of the report (if not contained in the report, readily available when needed);
    - date of the report, and time of release (if not contained in the report, readily available when needed);
    - page number to total number of pages (e.g. "Page 1 of 5", "Page 2 of 5", etc.).

5.9 Release of results

5.9.1 General

- The laboratory shall establish documented procedures for the release of examination results, including details of who may release results and to whom. The procedures shall ensure that the following conditions are met.
- When the quality of the primary sample received is unsuitable for examination, or could have compromised the result, this is indicated in the report.
- When examination results fall within established "alert" or "critical" intervals:
  — a physician (or other authorized health professional) is notified immediately [this includes results received on samples sent to referral laboratories for examination (see 4.5)];
  — records are maintained of actions taken that document date, time, responsible laboratory staff member, person notified and examination results conveyed, and any difficulties encountered in notifications.
- Results are legible, without mistakes in transcription, and reported to persons authorized to receive and use the information.
- When results are transmitted as an interim report, the final report is always forwarded to the requester.
- There are processes for ensuring that results distributed by telephone or electronic means reach only authorized recipients. Results provided orally shall be followed by a written report. There shall be a record of all oral results provided.
  - NOTE 1 For the results of some examinations (e.g. certain genetic or infectious disease examinations) special counselling may be needed. The laboratory should endeavour to see that results with serious implications are not communicated directly to the patient without the opportunity for adequate counselling.
  - NOTE 2 Results of laboratory examinations that have been separated from all patient identification may be used for such purposes as epidemiology, demography or other statistical analyses.
- See also 4.9.

5.9.2 Automated selection and reporting of results

- If the laboratory implements a system for automated selection and reporting of results, it shall establish a documented procedure to ensure that:
  - the criteria for automated selection and reporting are defined, approved, readily available and understood by the staff;
    - NOTE Items for consideration when implementing automated selection and reporting include changes from previous patient values that require review and values that require intervention by laboratory personnel, such as absurd, unlikely or critical values.
  - the criteria are validated for proper functioning before use and verified after changes to the system that might affect their functioning;
  - there is a process for indicating the presence of sample interferences (e.g. haemolysis, icterus, lipaemia) that may alter the results of the examination;
  - there is a process for incorporating analytical warning messages from the instruments into the automated selection and reporting criteria, when appropriate;
  - results selected for automated reporting shall be identifiable at the time of review before release and include date and time of selection;
  - there is a process for rapid suspension of automated selection and reporting.
- Revised reports
  - When an original report is revised there shall be written instructions regarding the revision so that:
    - the revised report is clearly identified as a revision and includes reference to the date and patient's identity in the original report;
    - the user is made aware of the revision;
    - the revised record shows the time and date of the change and the name of the person responsible for the change;
    - the original report entries remain in the record when revisions are made.
    - Results that have been made available for clinical decision making and revised shall be retained in subsequent cumulative reports and clearly identified as having been revised.
    - When the reporting system cannot capture amendments, changes or alterations, a record of such shall be kept.
